# Evaluation of the predictors and frequency of silent hypoxemia in COVID‐19 patients and the gap between pulse oximeter and arterial blood gas levels: A cross‐sectional study

**DOI:** 10.1002/hcs2.98

**Published:** 2024-06-17

**Authors:** Mohammad Javad Fallahi, Fatemehsadat Pezeshkian, Keivan Ranjbar, Rojan Javaheri, Reza Shahriarirad

**Affiliations:** ^1^ Department of Internal Medicine Shiraz University of Medical Sciences Shiraz Iran; ^2^ Thoracic and Vascular Surgery Research Center Shiraz University of Medical Science Shiraz Iran; ^3^ Student Research Committee Shiraz University of Medical Sciences Shiraz Iran; ^4^ School of Medicine Shiraz University of Medical Sciences Shiraz Iran

**Keywords:** COVID‐19, hypoxia, dyspnea, oximetry, O_2_ Saturation

## Abstract

**Background:**

Silent hypoxemia is when patients do not experience breathing difficulty in the presence of alarmingly low O_2_ saturation. It could cause rapid deterioration and higher mortality rates among patients, so prompt detection and identifying predictive factors could result in significantly better outcomes. This study aims to document the evidence of silent hypoxemia in patients with COVID‐19 and its clinical features.

**Methods:**

A total of 78 hospitalized, nonintubated patients with confirmed COVID‐19 infection were included in this study. Their O_2_ saturation was measured with a pulse oximeter (PO), and arterial blood gas (ABG) was taken. Demographic and clinical features were recorded. The Borg scale was used to evaluate dyspnea status, and patients with a score of less than two accompanied by O_2_ saturation of less than 94% were labeled as silent hypoxic. Univariate analysis was utilized to evaluate the correlation between variables and their odds ratio (OR) and 95% confidence interval (CI).

**Results:**

Silent hypoxemia was observed in 20 (25.6%) of the participants. The average difference between the PO and ABG methods was 4.36 ± 3.43. Based on regression analysis, dyspnea and respiratory rate demonstrated a statistically significant correlation with the O_2_ saturation difference between PO and ABG (OR: 2.05; *p* = 0.026; 95% CI: 0.248–3.847 and OR: 0.144; *p* = 0.048, 95% CI: 0.001–0.286). Furthermore, the Borg scale (OR: 0.29; *p* = 0.009; 95% CI: 0.116–0.740) had a significant reverse correlation with silent hypoxia.

**Conclusions:**

Silent hypoxemia can be a possible complication that affects some COVID‐19 patients. Further care should be bestowed upon the younger population and those with underlying neurological or mental illnesses. Furthermore, the respiratory rate, pulse oximeter, and arterial blood gas O_2_ levels should be considered alongside each other.

## Graphical abstract

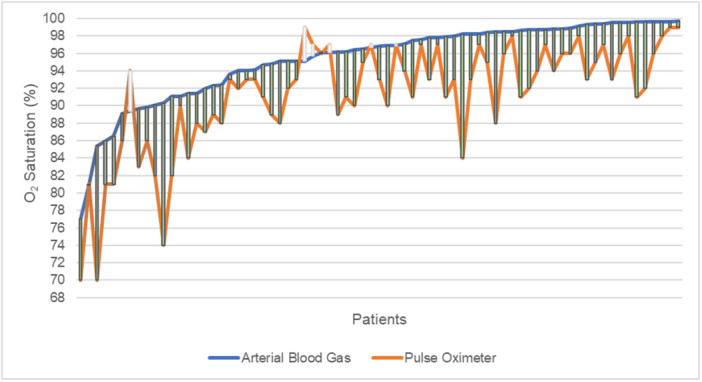

**Abbreviations**
ABG: arterial blood gas
COPD: chronic obstructive pulmonary disease
COVID‐19: coronavirus disease of 2019
ICH: International Council for Harmonization of Technical Requirements for Pharmaceuticals for Human Use
PCR: polymerase chain reaction
pH: potential of hydrogen
PO: pulse oximetry
SPSS: The Statistical Package for the Social Sciences

## 1 INTRODUCTION

In December 2019, an enveloped positive‐sense Ribonucleic acid virus from the family of Coronaviridae [1], later named Severe acute respiratory syndrome coronavirus 2 (SARS‐CoV‐2), initiated a global pandemic on March 11, 2020 [2]. By the date of this study, there have been 550,218,992 confirmed cases of coronavirus disease 2019 (COVID‐19) globally, including 6,343,783 deaths [3]. The clinical features of COVID‐19 vary from asymptomatic to severe progressive pneumonia, multiorgan dysfunction, and death [4, 5, 6]. The rising inquiry is how asymptomatic patients or those with mild symptoms with no perceivable respiratory discomfort can deteriorate swiftly and seemingly without warning into severe respiratory failure needing ventilator assistance [7].

Numerous case studies have depicted an unusual manifestation of COVID‐19: the development of hypoxia that is out of proportion to the patient's prodromes, a phenomenon called “silent hypoxemia.” This can occur in several situations. For instance, one study scrutinizes the existence of unapparent hypoxemia in casualties with wounded limbs [8]. Most recently, it has made headlines due to the large number of incidences of this occurring in individuals diagnosed with COVID‐19 [7, 9]. According to the normal physiology of the respiratory system, hypoxemia (SpO_2_ < 90%) results in dyspnea by stimulating the carotid bodies. However, the contradiction of this pattern is observed in several COVID‐19 patients who do not appear to be short of breath and have no complaints of breathing discomfort with allegedly normal physical exam (no signs of tachypnea, muscle retraction, or use of accessory muscles) despite their low level of O_2_ saturation detected on pulse oximetry (PO) [7, 9, 10]. Several studies have declared that the prevalence of silent hypoxemia varies from 20% to 40% of COVID‐19 patients [11]. As silent hypoxemia could result in rapid deterioration of COVID‐19 patients in a span of a few hours, investigating its causes and prevalence could help expect and manage this condition better. By this acknowledgment, prehospital PO can be used for early detection of silent hypoxemia in COVID‐19 patients for more efficient management [9]. However, the pathophysiology of this condition is yet to be wholly understood, and this calls for further investigation into silent hypoxemia and possible mechanisms [11].

The primary purpose of this study is to document the evidence of silent hypoxemia in COVID‐19 patients in a referral center in southern Iran by observing the inconsistency and variation among arterial blood gas (ABG) hypoxia, PO hypoxia, and patients' presentation to identify non‐clinically obvious hypoxemia and contribute to promoting patients' management protocols.

## 2 MATERIALS AND METHODS

### 2.1 Study design

Hospitalized patients with confirmed COVID‐19 were included in this study with primary criteria of polymerase chain reaction (PCR) documented SARS‐CoV‐2 carriage in a nasopharyngeal sample at admission. Patients were excluded if they depended on mechanical ventilation.

Before being included in the study, patients meeting inclusion criteria consented to participate. Written informed signed consent was obtained from adult participants (≥18 years) or parents or legal guardians for minors (<18 years). An information document that indicates the risks and benefits associated with participation in the study was given to each patient. Patients received information about their clinical status during care regardless of whether they decided to participate in the study. Regarding patient identification, a study number was assigned sequentially to included participants according to the range of patient numbers allocated to each study center. The study was conducted following the International Council for Harmonization of Technical Requirements for Pharmaceuticals for Human Use (ICH) guidelines of good clinical practice, the Helsinki Declaration, and applicable standard operating procedures and also approved by the Ethics Committee of Shiraz University of Medical Sciences (IR.SUMS.MED.REC. 1400.05).

### 2.2 Procedure

For the aim of the study, patients were evaluated based on their clinical symptoms, underlying disease, laboratory data, and imaging, considering their O_2_ saturation detected by PO in comparison with VBG.

A detailed data sheet including all patients' epidemiological, clinical, laboratory, and radiographic manifestations (based on chest X‐ray or computed tomography (CT) scan) with their O_2_ saturation (PO and VBG) and therapeutic approaches were collected.

### 2.3 Study participants and data collection

For this cross‐sectional study, a group of COVID‐19 patients who were admitted to COVID wards of Faghihi Hospital in Shiraz, Iran, in the years 2020–2021 were selected using a simple sampling method.

The sample size was calculated based on a study by Alhusain et al. [12] considering *α* = 0.05 (type 1 error) and *β*‐1 = 0.99 (power of the test); the number of 82 people was determined as the sample size.

The inclusion criteria were hospitalized patients with positive PCR documented SARS‐CoV‐2 carriage in a nasopharyngeal sample at admission or confirmed HRCT for COVID‐19, who were above 18 years of age, and who signed a consent written form.

The exclusion criteria were COVID‐19 patients who were intubated, had underlying pulmonary disease, were hypotensive, carried a peripheral vascular disease like Reynaud's disease, or wore nail polish or artificial nails, which could impair the accuracy of PO data.

The patients who met the above criteria were entered into this study. Their PO and ABG sampling were obtained simultaneously, in the way that standard PO was taken from both their index fingers, and the average was calculated and compared to other data. The data from PO and ABG sampling were put in for comparison. In addition, their clinical symptoms and imaging results were taken as well. It is worthy of notice that the ABG sampling is not a part of routine tests, and this test is obtained for study purposes only. If the data obtained from ABG sampling and PO did not correlate with the clinical symptoms of the patients at the time, then Silent hypoxemia was present.

To reach the patients' subjective state of dyspnea, the modified Borg scale was utilized to provide easy and quick information. It was rated from 0 to 10 to evaluate their dyspnea and is a reliable and valid tool for assessing the severity of dyspnea. Its validity has been proven over studies [13, 14] “Silent hypoxia” included those who reported no dyspnea (Borg scale of 0–2), even though oximetry saturation was <94%, were extracted.

### 2.4 Statistical analysis

The Statistical Package for the Social Sciences (IBM Corp. Released 2013. IBM SPSS Statistics for Windows; Version 22.0) was implemented for the data analysis. The baseline characteristics and demographic information were noted and reported in proportion and frequency (%). The *χ* ^2^ or Fisher's exact test was used for the evaluation of quantitative variables. Univariate analysis was utilized to evaluate the correlation between variables and their odds ratio (OR) and 95% confidence interval (CI). Furthermore, the Point Biserial and Pearson correlation was used to evaluate the correlation between the PO‐ABG O_2_ gap and the variables in our study. Regression model analysis was performed among variables with a *p* < 0.25 to assess for independent correlation with PO‐ABG O_2_ saturation gap through linear regression and for risk factors for silent hypoxia through logistic regression analysis. A *p *< 0.05 was considered statistically significant.

## 3 RESULTS

A total of 78 patients were evaluated in our study. The average age was 56.88 (SD: 16.21, range 19–91) years, and 42 (53.2%) were male, while 37 (46.8%) were female patients. Only 24 (30.4%) had no physical or mental comorbidity history.

Furthermore, 45 (57.0%) had a Borg scale of under two, while 20 also had an oximetry saturation of under 94%, which classified these patients as “silent hypoxia” [15]. The association between the features of the patients in our study and silent hypoxia is demonstrated in Table 1.

**Table 1:** Demographic and clinical features of coronavirus 2019 patients.

		Silent hypoxia			Association with PO‐ABG gap
Variable	Total; *N* = 78	Positive; *n* = 20	Negative; *n* = 58	*p* value; OR (95% CI)^a^	*p* value; correlation (95% CI)^b^
Age (yr); mean ± SD	56.9 ± 16.2	57.4 ± 16.3	56.7 ± 16.3	0.88; 1.00 (0.97–1.04)	0.19; −0.16 (−0.37 to 0.08)
Gender; *n* (%)					
Male	42 (53.8)	12 (60.0)	30 (51.7)	0.52; 0.71 (0.25–2.01)	0.47; −0.09 (−0.31 to 0.15)
Female	37 (46.2)	8 (40.0)	28 (48.3)		
Comorbid disease; *n* (%)					
Cardiovascular	22 (27.8)	4 (20.0)	18 (31.0)	0.34; 0.56 (0.16–1.90)	0.80; 0.03 (−0.20 to 0.26)
Endocrinological	21 (26.9)	3 (15.0)	18 (31.0)	0.16; 0.39 (0.10–1.51)	0.78; 0.03 (−0.20 to 0.26)
Neurological	7 (9.0)	4 (20.0)	3 (5.2)	0.07; 4.58 (0.93–22.64)	0.41; −0.98 (−0.32 to 0.14)
Pulmonary	3 (3.8)	0 (0.0)	3 (5.2)	0.57; –	0.92; 0.01 (−0.22 to 0.24)
Mental	2 (2.6)	2 (10.0)	0 (0)	0.06; –	0.60; −0.06 (−0.29 to 0.17)
None	24 (30.8)	8 (40.0)	16 (27.6)	0.30; 1.75 (0.60–5.07)	0.26; −0.13 (−0.35 to 0.10)
Symptoms; *n* (%)					
Dyspnea	60 (76.9)	16 (80.0)	44 (75.9)	1.00; 1.27 (0.36–4.44)	**0.02; −0.14 (−0.36 to 0.10)**
Cough	47 (60.3)	9 (45.0)	38 (65.5)	0.11; 0.43 (0.15–1.21)	0.49; −0.08 (−0.31 to 0.15)
Fever	39 (50.0)	11 (55.0)	28 (48.3)	0.60; 1.31 (0.47–3.63)	0.47; −0.09 (−0.31 to 0.15)
Body pain	36 (46.2)	8 (40.0)	28 (48.3)	0.52; 0.71 (0.25–2.01)	0.38; 0.11 (−0.13 to 0.33)
Malaise	36 (46.2)	11 (55.0)	25 (43.1)	0.36; 1.61 (0.58–4.49)	0.87; −0.02 (−0.21 to 0.25)
Anorexia	21 (26.9)	7 (35.0)	14 (24.1)	0.35; 1.69 (0.56–5.08)	0.39; −0.10 (−0.32 to 0.13)
Gastrointestinal	9 (11.5)	1 (5.0)	8 (13.8)	0.43; 0.33 (0.39–2.81)	0.24; −0.14 (−0.36 to 0.10)
Headache	9 (11.5)	2 (10.0)	7 (12.1)	1.00; 0.81 (0.15–4.27)	0.21; 0.15 (−0.09 to 0.37)
Sore throat	4 (5.1)	3 (15.0)	1 (1.7)	0.050; 10.06 (0.98–103.1)	0.62; −0.06 (−0.29 to 0.17)
Decrease level of conciseness	1 (1.3)	0 (0)	1 (1.7)	1.00; –	**0.03; 0.25 (0.018–0.45)**
Dizziness	1 (1.3)	0 (0)	1 (1.7)	1.00; –	0.80; 0.03 (−0.20 to 0.26)
Respiratory rate; mean ± SD	23.0 ± 5.4	21.6 ± 4.8	23.5 ± 5.6	0.18; 0.91 (0.80–1.04)	**0.02; −0.27 (−0.47 to −0.05)**
pH; mean ± SD	7.5 ± 0.1	7.4 ± 0.1	7.5 ± 0.1	0.51; 0.03 (0.00–1020.4)	**0.02; −0.27 (−0.47 to −0.04)**
PaCO_2_; mean ± SD	31.1 ± 6.7	30.9 ± 6.3	31.1 ± 6.9	0.87; 0.99 (0.92–1.07)	0.57; 0.07 (−0.17 to 0.29)
HCO_3_; mean ± SD	22.3 ± 4.5	22.2 ± 2.3	22.3 ± 5.0	0.94; 1.00 (0.89–1.12)	0.96; −0.01 (−0.24 to 0.22)
PaO_2_; mean ± SD	98.1 ± 47.5	81.4 ± 25.0	103.9 ± 52.4	0.08; 0.99 (0.97–1.00)	0.60; 0.06 (−0.20 to 0.29)
BORG scale; *n* (%) or median [Q1–Q3]					
Nothing (0)	18 (23.1)	7 (35.0)	11 (19.0)	0.22; 2.30 (0.74–7.12)	0.28; 0.13 (−0.11 to 0.35)
Very very low (0.5)	13 (16.7)	5 (25.0)	8 (13.8)	0.30; 2.08 (0.59–7.33)	0.13; 0.18 (0.05–0.39)
Very low (1)	14 (17.9)	8 (40.0)	6 (10.3)	**0.006; 5.78 (1.69–19.78)**	0.13; 0.18 (−0.05 to 0.39)
Low (2)	8 (10.3)	0 (0)	8 (13.8)	0.11; –	0.35; −0.11 (−0.33 to 0.12)
Moderate (3)	4 (5.1)	0 (0)	4 (6.9)	0.57; –	0.71; 0.05 (−0.19 to 0.27)
Somewhat high (4)	6 (7.7)	0 (0)	6 (10.3)	0.33; –	0.58; −0.07 (−0.17 to 0.29)
High (5, 6)	12 (15.4)	0 (0)	12 (20.7)	**0.03**; –	0.06; −0.22 (−0.43 to 0.01)
Very high (7–9)	3 (3.9)	0 (0)	3 (5.1)	0.57; –	0.46; −0.09 (−0.31 to 0.15)
Maximal exertion (10)	0 (0)	0 (0)	0 (0)	–	–
Total; median [Q1–Q3]	1 [0.5–4]	0.5 [0–1]	2 [0.5–5]	**0.005; 0.38 (0.20–0.75)**	0.07; −0.22 (−0.42 to 0.02)

*Note*: Bold values indicator of significant association.

Abbreviations: ABG, arterial blood gas; CI, confidence interval; OR, odds ratio; PO, pulse‐oximeter.

^a^ *χ* ^2^/Fisher's exact test.

^b^ Point Biserial correlation/Pearson's correlation.

**Figure 1:**
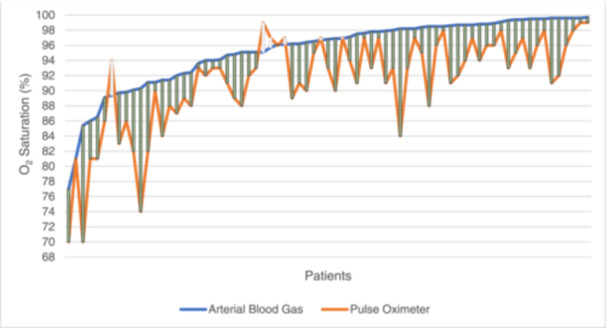
O_2_ saturation levels based on the pulse oximeter (PO) and arterial blood gas (ABG) values. Each point in the *x*‐axis represents a patient. Bar values indicate the gap between the values in which green bars represent higher ABG O_2_ compared to PO O_2_, while white represents higher PO O_2_ compared to ABG O_2_.

The average O_2_ saturation of the patients based on PO was 91.45 ± 6.20 (range 70.0–99.0), while based on ABG evaluation was 95.33 ± 4.60 (range 77.0–99.7). The average difference between the two methods was 4.36 ± 3.43 (Figure 1). The correlation between the features in our study and the PO‐ABG gap is demonstrated in Table 1. There was no significant association between the PO‐ABG gap and silent hypoxia in our study (for silent vs. nonsilent hypoxia: −5.46 vs. −3.56; *p* = 0.09; 95% CI: −0.35 to 4.16).

As demonstrated in Table 1, there was a significant association between the Borg scale and silent hypoxia, in which silent hypoxia was significantly more observed in the very low Borg scale (*p* = 0.006; OR: 5.8) and significantly less observed in the high Borg scale (*p* = 0.030).

Furthermore, there was a statistical association between dyspnea and the O_2_ saturation difference between the PO and ABG, in which the gap between the two methods increased in patients with dyspnea (*r* = −0.14, *p* = 0.02). Higher pH levels and respiratory rate were also associated with a lower gap between our patients' PO and ABG O_2_ levels (*r* = −0.266 and −0.274, *p* = 0.023 and 0.019).

**Figure 2:**
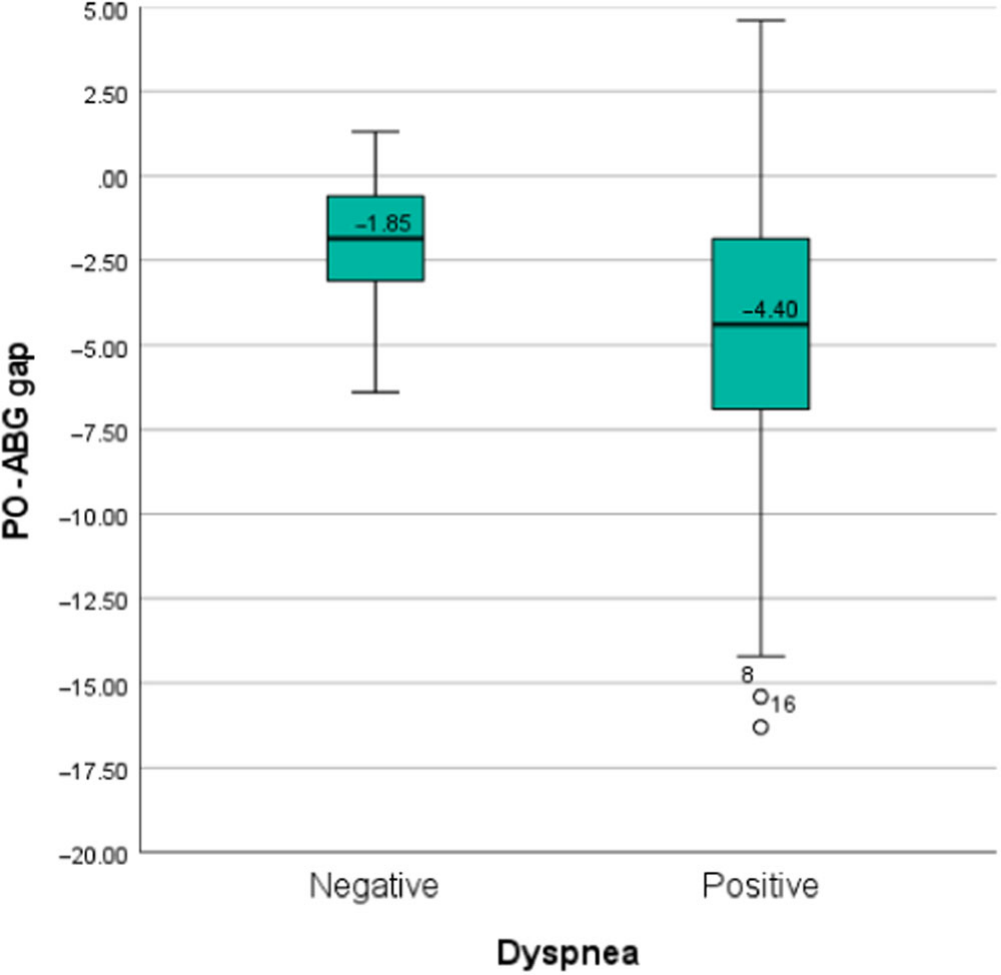
Box plot of pulse‐oximeter and atrial blood gas O_2_ gap between patients with and without dyspnea among coronavirus patients. *p* = 0.026; 95% confidence interval: 0.248–3.847.

**Figure 3:**
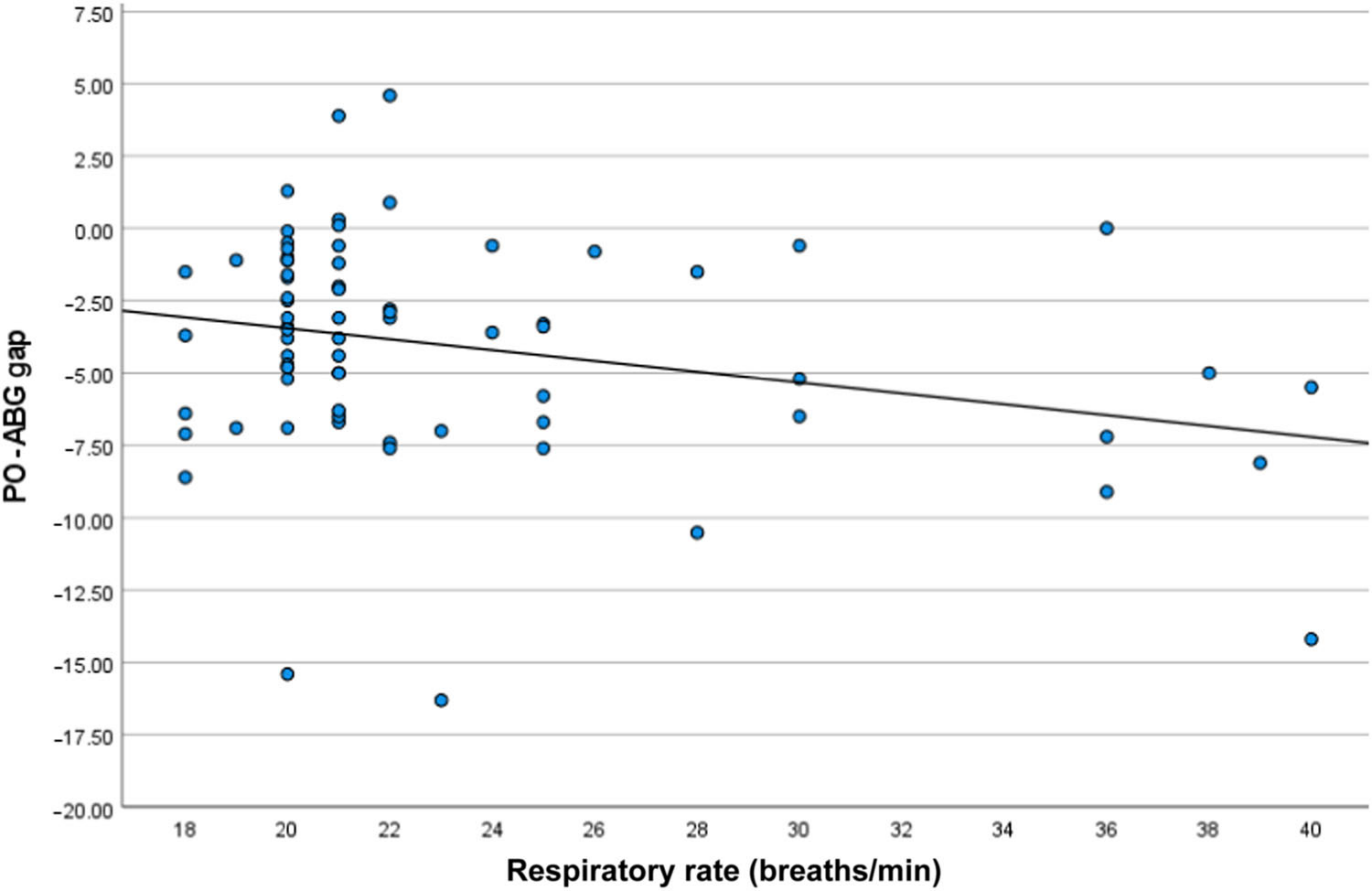
Scatter plot of pulse‐oximeter and atrial blood gas O_2_ gap based on respiratory rate among coronavirus patients. *p* = 0.02; correlation coefficient: −0.27; 95% confidence interval: −0.47 to −0.05.

After evaluating our data based on the linear regression model, only dyspnea and respiratory rate demonstrated a statistically significant correlation with the O_2_ saturation difference between PO and ABG (OR: 2.05; *p* = 0.026; 95% CI: 0.248–3.847 and OR: 0.144; *p* = 0.048, 95% CI: 0.001–0.286). Figures 2 and 3 demonstrate the box plot of these variables based on the PO‐ABG gap.

**Figure 4:**
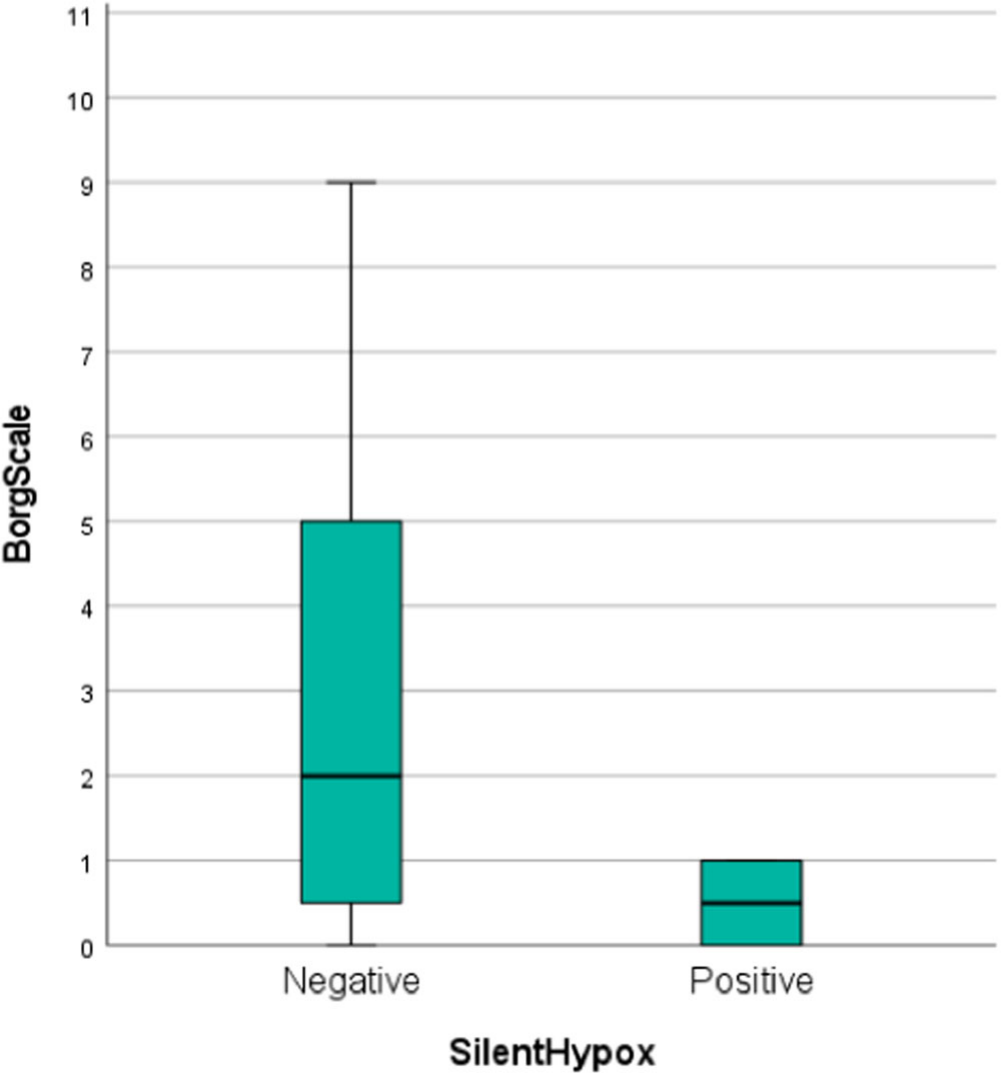
Box plot of Borg scale based on silent hypoxia among coronavirus patients. *p* = 0.009; 95% confidence interval: 0.116–0.740.

Furthermore, based on logistic regression analysis, the Borg scale (OR: 0.29; *p *= 0.009; 95% CI: 0.116–0.740) had a significant reverse correlation with silent hypoxia (Figure 4).

## 4 DISCUSSION

Silent hypoxemia is the condition in which patients do not experience any breathing difficulties in the presence of an alarmingly low saturation of O_2_ [11]. As patients with silent hypoxemia are prone to deteriorate suddenly and are usually undiagnosed until the late stages of their disease, recognizing predictable factors and their overall prevalence plays a crucial role in their management. Furthermore, the accuracy of PO and ABG data was also compared to obtain the most reliable route for obtaining O_2_ saturation. Silent hypoxemia was observed in 25% of our participants, and the gap between PO and ABG had a positive association with age and dyspneic patients; in contradiction, their gap had a reverse association with ABG potential of hydrogen (pH) levels and Borg scale.

The primary objective of this study was to document evidence of silent hypoxemia in COVID‐19 patients and explore its clinical features. Silent hypoxemia is associated with rapid deterioration, like developing pneumonia or respiratory decompensation, along with higher mortality rates [16]. This emphasizes the value of timely therapeutic interventions to identify silent hypoxemia, such as supplemental oxygen therapy or other appropriate interventions tailored to individual patient needs. Lei and colleagues demonstrated the significance of immunological biomarkers, and also the importance of timely intervention in the context of point‐of‐care testing, providing a parallel example of the impact of early detection on patient outcomes [17, 18]. Various tools like the 4C scores have been developed and advocated by WHO guidelines that stratify the mortality risk in COVID‐19 patients by recording baseline demographics along with the O_2_ profile [16, 19, 20]. Integrating additional diagnostic tools into the routine assessment of COVID‐19 patients could enhance the early detection of silent hypoxemia [21].

Several studies have estimated the prevalence of silent hypoxemia in COVID‐19 patients to be around 20%–40%, which was in line with our findings [11]. COVID‐19 patients were also observed to demonstrate silent hypoxemia more than non‐COVID‐19 patients [22]. To provide comprehensive awareness of the symptoms that are the culprits of this phenomenon, possible symptoms, and underlying mechanisms were taken into account. Usually, lower O_2_ saturation is accompanied by an increase in respiratory rate. However, this finding was not evident in silent hypoxemic patients in different studies [11]. Although silent hypoxemic patients had a lower respiratory rate than nonsilent hypoxemic patients, the difference was not statistically significant in our study. In another study, patients suitable for discharge underwent a 6‐min walk test. It was found that 50% of them developed hypoxia without apparent dyspnea [23]. Overall, it is evident that silent hypoxemia is a prevalent ongoing phenomenon in COVID‐19 patients, and further investigations are in order.

Among the recorded symptoms, sore throat was significantly associated with silent hypoxemia. Moreover, cough was also related to silent hypoxemia, which was not statistically significant. In other studies, cough and fever were counted as predictive symptoms for silent hypoxemia [12]. Comorbid diseases were not observed to have a significant association with developing silent hypoxemia; however, mental and neurologic diseases had a noticeable yet statistically insignificant association with silent hypoxemia. Alhusain et al. conducted cardiac diseases as a predictive factor for silent hypoxemia, which did not align with our study findings.

Although some studies report comorbid diseases such as diabetes could contribute to the lack of O_2_, our findings indicate otherwise, which is also in line with other studies [15]. All in all, these results could implicate that further mechanisms beyond what we have found, such as COVID‐19‐induced damages to the nervous system or vasculature, may play a role in silent hypoxemia.

Several hypotheses regarding the underlying etiology of silent hypoxemia have been made. One report states corticolimbic network neuronal damage, which could be caused by the COVID‐19 virus, could alter the perception of the control of respiration and dyspnea [24]. Autopsy brain specimens from deceased COVID‐19 patients revealed a neuronal loss in the cerebellar Purkinje cell layer, cerebral cortex, and hippocampus, along with encephalitis, stroke, and hemorrhage [11, 25]. Furthermore, inducing a lack of hypoxic vasoconstriction on the blood vessels, pulmonary microthrombosis, or fever has also been proposed [11, 15]. Lastly, none of these evidence have enough scientific proof to indicate the primary pathophysiology of silent hypoxemia.

To answer the inquiry of whether PO shows accurate O_2_ levels, the ABG O_2_ levels and PO O_2_ levels were compared and analyzed. PO was obtained from the fingertips of the index fingers in both hands. While keeping in mind that fingertips PO has been observed to exhibit a higher gap in relation to ABG than earlobe PO, the median difference between the two methods was 3.4, which was higher than what previous studies have observed [26, 27]. Over a number of studies, age was observed to have a reverse relation with the gap between PO and ABG [28]. This finding could indicate that more attention should be given to younger patients' O_2_ saturation levels so as not to miss silent hypoxemia since more severe presentations of the disease are mostly expected to occur in older patients [29] and younger individuals usually get neglected. Higher pH levels also coordinated with a lower gap between PO and ABG. Moreover, the respiratory rate had a linear relation with a lower gap between PO and ABG. As silent hypoxemic patients are observed to have lower respiratory rates, this could mean those with silent hypoxemia have a higher gap between PO and ABG [11].

Measuring the diversity between ABG and PO in chronic obstructive pulmonary disease (COPD) patients indicated that chronic lung diseases could affect the gap between ABG and PO. This could be because the pulse oximeter demonstrated the ratio of oxyhemoglobin to total hemoglobin in the pulsatile arterial vessels, and the factors that could influence the arteries' pulsatility could disturb PO accuracy [30]. Moreover, PO is unreliable in hypothermia, low perfusion phases, carbon monoxide, anemia, and icterus [31]. Comparing the PO and ABG measurements in noncritically ill COVID‐19 patients concluded that high ferritin and fibrinogen levels and low levels of lymphocytes could disturb PO accuracy [32]. Furthermore, the accuracy of different PO devices varies and may demonstrate to higher or lower presentation of the O_2_ status of the patient [33]. In conclusion, both PO and ABG should be taken into consideration, especially in patients who are more prone to exhibit this diversity, to achieve a better outcome.

Some limitations that should be addressed are as follows. First of all, this study was a single‐referral center design with a small number of participants, and more diverse studies are needed in the future. Second, since this study was conducted in only one span of time with the presumption of only limited virus variants, virus variants were not screened and taken into consideration. We also did not evaluate patients' biomarker concentrations during our study and their association with silent hypoxia. Moreover, we acknowledge the potential limitations of the Borg scale in measuring resting dyspnea. To address this, we propose a comprehensive approach, supplementing the Borg scale with additional assessment methods, including standardized questionnaires, patient interviews, and clinical observation. By adopting these complementary measures and ensuring transparent reporting of limitations in our manuscript, we aim to provide a more thorough and nuanced evaluation of the patient's respiratory status. Lastly, further clinical trials with more participants and more control should be carried out with a follow‐up to estimate silent hypoxemia's effect on prognosis.

## 5 CONCLUSION

The prevalence of silent hypoxemia in COVID‐19 patients is noticeable, and its early detection plays a crucial role in the prognosis of these patients. Further care should be bestowed upon the younger population and those with underlying neurological or mental illnesses. Furthermore, the respiratory rate should be taken into consideration along with both PO and ABG O_2_ levels to avoid misjudging critical cases.

## AUTHOR CONTRIBUTIONS

*Study conception and design*: Mohammad Javad Fallahi and Reza Shahriarirad. *Acquisition of data*: Fatemehsadat Pezeshkian and Rojan Javaheri. *Drafting and critical revision of the manuscript*: Fatemehsadat Pezeshkian, Keivan Ranjbar, and Reza Shahriarirad. *Analysis and interpretation of data*: Reza Shahriarirad. All authors read and approved the final version of the manuscript.

## CONFLICT OF INTEREST STATEMENT

The authors declare no conflict of interest.

## ETHICS STATEMENT

The study was approved by the Research Ethics Committee of the School of Medicine‐Shiraz University of Medical Sciences (Ethical code: IR.SUMS.MED.REC.1400.057). Permission to carry out the study and access patient records was sought from the respective university administrators, and the study was conducted in compliance in accordance with the relevant guidelines and regulations and the Declaration of Helsinki and was also approved by the ethics committee of the university.

## INFORMED CONSENT

Written informed consent for participation was obtained from the patients.

## Data Availability

The data sets used and analyzed during the current study are available from the corresponding author on reasonable request and with permission of the Research Ethics Committee of the School of Medicine‐Shiraz University of Medical Sciences.
